# Rethinking antibiotic prophylaxis in orthopaedic oncology: insights from a cohort study of endoprosthetic infections

**DOI:** 10.5194/jbji-10-33-2025

**Published:** 2025-02-26

**Authors:** Tariq Azamgarhi, Craig Gerrand, Simon Warren

**Affiliations:** 1 Pharmacy Department, Royal National Orthopaedic Hospital NHS Trust, Brockley Hill, Stanmore, HA7 4LP, UK; 2 Division of Orthopaedic Oncology, Royal National Orthopaedic Hospital NHS Trust, Sarcoma Unit, Brockley Hill, Stanmore, HA7 4LP, UK; 3 Bone Infection Unit, Royal National Orthopaedic Hospital NHS Trust, Brockley Hill, Stanmore, HA7 4LP, UK

## Abstract

**Introduction**: Endoprosthetic replacement (EPR) is the preferred limb salvage method for musculoskeletal tumours involving bone; however, infection rates range from 8 % to 12 %. We investigated the impact of antibiotic prophylaxis at primary implantation on the development of prosthetic joint infection (PJI). **Methods**: We conducted a retrospective analysis of patients who underwent primary EPRs between 2010 and 2021. Prosthetic joint infections were identified and classified according to criteria from the European Bone and Joint Infection Society (EBJIS). The follow-up period extended until an infection was identified, subsequent surgery for non-infectious reasons occurred or the last known follow-up was conducted. For all primary procedures, we collected details of postoperative complications at the surgical site, including superficial wound infections, delayed wound healing and wound dehiscence. PJIs were divided into two groups. The first group included patients with an uncomplicated postoperative course, while the second comprised those with either postoperative wound problems or infections from an identifiable source. **Results**: Out of 1064 patients, 73 (6.9 %) developed PJI within a median follow-up of 25.6 months (IQR 8.8–52.7). A total of 26 % of PJIs were attributed to primary implantation, while 74 % of PJIs were due to secondary causes, with 47 % having wound complications and 27 % presenting acutely. The microbiological profiles between groups differed significantly, with infections from skin flora related to primary implantation and a high proportion of other bacteria (Gram-negatives and enterococci) linked to secondary infections. **Conclusions**: Skin flora are likely responsible for infections related to the primary procedure, and antibiotic prophylaxis should be optimised accordingly. Additional measures are needed to prevent secondary infections.

## 1 Introduction

Endoprosthetic replacement (EPR) is an established method of limb salvage following wide resection of musculoskeletal tumours involving bone. Despite the benefits of EPR, such as flexible and predictable reconstruction with immediate weight-bearing, the risk of infection is high for reasons including the long operating times associated with complex procedures, extensive tissue dissection, immunocompromised patients, and large prostheses (Racano et al., 2013).

Antimicrobial prophylaxis is one of several high-impact measures that reduce infection risk. The PARITY trial demonstrated that extending the duration of antibiotic prophylaxis from 24 h to 5 d does not significantly alter the rates of surgical site infections (SSIs) and is associated with increased antibiotic-related complications, particularly *Clostridioides difficile*-associated colitis (Ghert et al., 2022). However, no consensus exists about the optimal regimen for prophylaxis in this group of patients, which guidelines recommend should cover organisms most likely to cause infection (Strony et al., 2019).

EPR infections can be acquired during the primary implantation procedure or secondarily, for example, after bacteraemia or wound complications. Establishing causality is challenging in cases where both wound complications and deep infection occur. Wound complications may predispose to deep infection, or alternatively, an evolving deep infection may manifest initially with wound problems. Despite this limitation, understanding infection patterns in patients with and without wound complications could help guide prevention strategies. For example, optimising surgical antibiotic prophylaxis may be key if most infections occur in patients without wound problems. Conversely, additional preventive measures beyond surgical prophylaxis would be needed if infections predominantly occur in patients with wound complications.

We hypothesised that infections occurring in patients without wound complications or other clear secondary causes are more likely to have been acquired during the primary procedure and, therefore, are potentially preventable through optimised surgical prophylaxis. Conversely, infections in patients with wound complications may require additional preventive interventions.

Our objectives were to (1) determine the proportion of prosthetic joint infections (PJIs) that could reasonably be attributed to primary implantation and (2) analyse their microbiological profiles to guide prophylaxis choice.

## 2 Methods

We conducted a retrospective review of clinical records for all primary EPRs implanted for musculoskeletal tumours at our hospital from January 2010 to September 2022. Patients were identified using a prospectively maintained EPR register based on hospital coding validated by implant register records.

Data were collected from electronic inpatient and outpatient records. Data included demographics (age and sex), tumour type and site, radiotherapy or chemotherapy, BMI, comorbidities, duration of surgery, details of any reoperations to the primary EPR, and antibiotic prophylaxis administered. In September 2013, we changed the first-line antibiotic regimen for surgical prophylaxis in total joint arthroplasty (TJA) from cefuroxime (1.5 g every 8 h for three doses, with the first dose at induction) to single doses of teicoplanin (10 mg kg^−1^) and gentamicin (5 mg kg^−1^) at induction.

Patients were divided into two groups based on the presence or absence of postoperative wound complications. Wound complications were defined as any superficial wound infection according to UKHSA criteria, documented delay of wound healing, seroma, haematoma formation or wound dehiscence (Harrington et al., 2013).

### Prosthetic joint infection

Patients were routinely followed up within 3 months and at least annually subsequently. All suspected prosthetic joint infections (PJIs) were assessed and graded as likely or confirmed according to diagnostic criteria from the European Bone and Joint Infection Society (EBJIS) (McNally et al., 2023).

All infections were reviewed and classified based on whether they were likely acquired during the primary surgery or as a secondary infection. Infections were considered secondary if there was an identifiable cause, such as a preceding infection at another site or a wound complication occurring around the time of surgery. Infections that did not fit these criteria were attributed to the surgical procedure and were, therefore, considered potentially influenced by antibiotic prophylaxis during that surgery.

Additionally, infections were categorised by the time elapsed since implantation: early (within 3 months), delayed (3 to 24 months), and late (beyond 24 months).

In our unit, standard practice for patients with EPR infections involves returning to theatre for an open procedure involving deep sampling of synovial fluid and/or periprosthetic tissue. Patients who do not have surgery undergo a radiologically guided deep aspirate before starting antibiotic suppression therapy. All samples were sent for microbiological analysis. Tissue and fluid samples were processed using a bead mill in a class-2 safety cabinet, inoculated into BACTEC^®^ bottles and incubated for up to 14 d. If the bottles flagged positive, they were sub-cultured onto solid media, and organisms were identified using matrix-assisted laser desorption ionisation time-of-flight (MALDI-TOF) mass spectrometry.

For diagnostic purposes, a minimum of two culture-positive deep samples was necessary for identifying low-virulence pathogens such as coagulase-negative staphylococci (CoNS) and *Cutibacterium acnes*. Conversely, identifying more virulent pathogens, including *Staphylococcus aureus*, beta haemolytic streptococci or Gram-negative bacilli, required at least one positive culture. This approach is in accordance with the recommendations from the Infectious Diseases Society of America guidelines (Osmon et al., 2013).

We categorised pathogens into two groups: primary flora, which included *Streptococcus* spp., *Staphylococcus aureus*, coagulase-negative staphylococci (CoNS) and other skin flora, and secondary flora, which comprised *Enterococcus* spp. and Gram-negative bacilli, which may be selected by antibiotic use. The organism profiles from primary and secondary infection groups were compared.

## 3 Statistical analysis

Descriptive statistics were used to estimate frequencies of the study variables, which were described as counts (percentage) for dichotomous values and the median (interquartile range, IQR) for continuous values. Categorical data on baseline characteristics were compared using a two-sided Pearson $\chi^{2}$ test or a Fisher exact test. Continuous variables, expressed as median (IQR), were compared using Student's t test or a Mann–Whitney U test.

We applied survival analysis to investigate the impact of wound complications on PJI over time. The outcome variable, time to likely or confirmed PJI according to published criteria, was constructed as the time between index surgery and the diagnosis of PJI. Patients receiving a reoperation for non-infectious reasons or who were infection-free at their last follow-up were considered censored in the analysis. The Kaplan–Meier method was used to plot cumulative hazards for the wound and non-wound groups.

All analyses were performed using SPSS 17.0 (IBM SPSS, Chicago, IL, USA).

## 4 Results

The study included 1064 patients; 104 (9.8 %) experienced a wound complication (Table 1). A total of 73 (6.9 %) PJIs were reported over a median follow-up period of 25.6 months (IQR 8.8–52.7): 38 of 104 (36.5 %) in the group with wound complications and 35 of 960 (3.6 %) in the group without wound complications.

**Figure 1:**
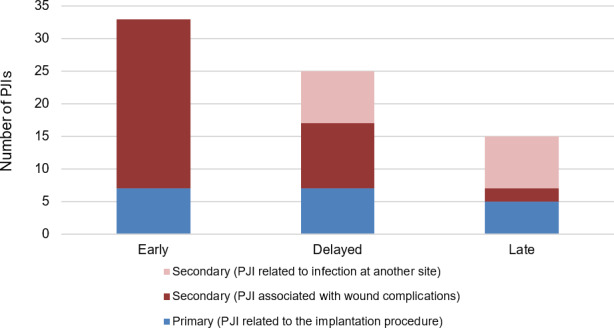
Bar chart of PJIs according to timing of presentation and whether the infection is reasonably attributable to primary surgery or secondary causes.

Figure 1 shows the proportion of PJIs according to timing of presentation and likely aetiology. In the group without wound complications, a total of 16 PJIs were associated with infections at another site, none of whom experienced initial wound complications. Thus, 19 (26.0 %) of the remaining PJIs could be directly linked to primary implantation.

Patients who had wound complications were more likely to have osteosarcoma compared with other tumour types (33.7 % vs. 24.0 %, p=0.040 ), EPRs involving the pelvis (12.5 % vs. 2.6 %, p<0.001 ) and tibia (23.1 % vs. 12.0 %, p=0.002 ), longer procedure times (median duration 3.5 h, IQR 2.3–4.8 vs. 2.4 h, IQR 1.9–3.1), and concurrent hypertension (41.3 % vs. 26.8 %, p=0.003 ). No significant differences were observed in BMI, other comorbidities or the use of radiotherapy.

**Figure 2:**
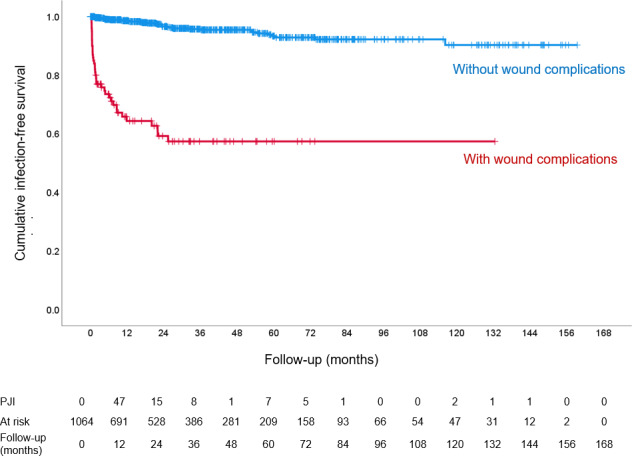
Cumulative probability of being free of PJI within EPR patients with (red) and without (blue) wound problems at primary surgery.

Survival analysis indicated that infections in the group with wound complications predominantly occurred within the first year after primary implantation, with survival curves diverging for up to 3 years (Fig. 2). Wound complication exhibited a statistically significant increased risk for PJI (hazard ratio (HR) 14.47, 95 % confidence interval (CI) 9.04–23.15, p<0.001 ).

Microbiological cultures were obtained for all 73 cases (Table 2); 11 were culture-negative infections. Among the 62 culture-positive cases, 88 organisms were isolated, most commonly staphylococci (49.4 %), CoNS (30.3 %) and *Staphylococcus aureus* (19.1 %), followed by Gram-negative bacilli (23.6 %), streptococci (15.7 %), enterococci (5.6 %) and other skin flora (5.6 %).

The microbiological profiles significantly differed between groups, with a higher proportion of infections related to primary implantation caused by primary pathogens and skin flora. In contrast, Gram-negative organisms were predominantly associated with PJIs in the wound complication group (37.0 % vs. 0 %, p=0.004 ). Further analysis of the 16 isolates linked to primary implantation showed that two of them (12.5 %) would not have been covered by the antibiotic prophylaxis administered during the primary EPR procedure.

**Table 1:** Baseline characteristics of wound and non-wound groups.

Characteristics	No wound complication	Wound complications	P value
	N=960	N=104	
Median (IQR) age (year)	53 (23–67)	53 (19–69)	0.889
No. (%) of patients by sex			
Female	463 (48.6)	46 (44.2)	0.438
Male	497 (51.8)	58 (55.8)	
No. (%) of patients with the following BMI:			
<30 kg m^−2^	817 (85.1)	83 (79.8)	0.201
30– <35 kg m^−2^	124 (12.9)	20 (19.2)	0.102
≥35 kg m^−2^	9 (2.0)	1 (1.0)	1.000
No. (%) of patients with the following comorbidity:			
hypertension	257 (26.8)	43 (41.3)	**0.003**
diabetes mellitus	87 (9.1)	10 (9.6)	0.995
liver disease	25 (2.6)	5 (4.8)	0.205
lung disease	187 (19.5)	21 (20.2)	0.965
chronic kidney disease	25 (2.6)	5 (4.8)	0.205
rheumatoid arthritis	9 (0.9)	2 (1.9)	0.293
Chemotherapy:			
yes	444 (46.2)	47 (45.2)	0.837
no	516 (53.8)	57 (54.9)	
Radiotherapy:			
yes	200 (20.8)	19 (18.3)	0.539
no	760 (79.2)	85 (81.7)	
Median (IQR) op duration (hours)	2.4 (1.9–3.1)	3.5 (2.3–4.8)	<0.001
No. (%) endoprosthesis type:			
upper limb	184 (19.2)	10 (9.6)	**0.024**
pelvis	25 (2.6)	13 (12.5)	<0.001
proximal femur	307 (32.0)	20 (19.2)	**0.010**
mid-femur or total femur	50 (5.2)	10 (9.6)	0.104
distal femur	279 (29.1)	27 (26.0)	0.583
tibia	115 (12.0)	24 (23.1)	**0.002**
No. (%) tumour type:			
osteosarcoma	230 (24.0)	35 (33.7)	**0.040**
chondrosarcoma	131 (13.6)	16 (15.4)	0.735
Ewing's	60 (6.3)	4 (3.8)	0.446
spindle cell	34 (3.5)	3 (2.9)	1.000
pleomorphic sarcoma	24 (2.5)	5 (4.8)	0.194
leiomyosarcoma	14 (1.5)	0 (0)	0.384
other sarcoma	36 (3.8)	8 (7.7)	0.067
lymphoma/myeloma	31 (3.2)	2 (1.9)	0.764
metastatic bone disease	400 (41.7)	31 (29.8)	**0.025**

Bold values denote statistical significance at the p<0.05 level. Column percentages may not sum to 100 % due to rounding.

In the survival analysis, 1064 cases were included, with 73 censored due to infection as the outcome. Of the remaining 991 patients, 849 were censored at the last follow-up without infection or reoperation. The remaining 142 cases involved reoperations for non-infectious causes: 75 cases were revisions, 40 were reoperations and 27 resulted in amputation.

**Table 2:** Summary of organisms isolated in complicated and uncomplicated groups. Bold values denote statistical significance at the p<0.05 level.

Organism group	Organisms isolated	Primary (associated with	Secondary (related	Breakdown of secondary causes		P value*
	N=88 (%)	implantation procedure)	to other causes)			
				Wound	Infection at	
				problems	another site	
Primary flora						
*Streptococcus* spp.	14 (15.9)	1 (6.2 %)	13 (18.1 %)	4 (7.4 %)	9 (50.0 %)	0.450
*Staphylococcus aureus*	16 (18.2)	7 (43.8 %)	9 (12.5 %)	5 (9.3 %)	4 (22.2 %)	**0.008**
Coagulase-negative staphylococci	27 (30.7)	7 (43.8 %)	20 (27.8 %)	17 (31.5 %)	3 (16.7 %)	0.239
Other skin flora	5 (5.7)	1 (6.2 %)	4 (5.6 %)	3 (5.6 %)	1 (5.6 %)	1.000
Secondary flora						
Gram-negative bacilli	21 (23.9)	0 (0.0 %)	21 (29.2 %)	20 (37.0 %)	1 (5.6 %)	**0.010**
*Enterococcus* spp.	5 (5.7)	0 (0.0 %)	5 (6.9 %)	5 (9.3 %)	0 (0.0 %)	0.580

* Comparing rates of intraoperatively acquired and total acquired by secondary causes. Column percentages may not sum to 100 % due to rounding.

## 5 Discussion

This large cohort study investigated the impact of antibiotic prophylaxis on the development of PJIs in EPRs implanted to treat musculoskeletal tumours. An in-depth analysis was conducted to identify PJIs that could reasonably be attributed to primary implantation and are, therefore, potentially preventable by antibiotic prophylaxis.

We found that 19 of 73 (26 %) were related to primary implantation. Conversely, 74 % were reasonably attributed to secondary causes, with 47 % of PJIs occurring in patients with wound complications and a further 27 % presenting following an infection at another site and, therefore, are likely unrelated to primary implantation. Wound complications emerged as the most frequently identified secondary cause of EPR infection. A comparison of the characteristics of patients with and without wound complications identified significant differences in patient, oncology and surgery-related factors. Wound complications were significantly more common in surgery involving the tibia and pelvis, supporting findings from several studies that identified higher infection rates at these sites compared to the upper limbs and femur (Morii et al., 2010; Peel et al., 2014).

Survival analysis indicated that patients with wound complications had a significantly higher risk of developing PJI (HR 14.47, 95 % CI 9.04–23.15, p<0.001 ), especially for early-onset polymicrobial PJIs caused by Gram-negative bacteria. This is consistent with previous studies showing wound complications to be an important factor in developing PJI (10–13). The effectiveness of antibiotic prophylaxis is limited in these situations, as infections are less likely to be acquired during primary implantation. Hence, additional measures such as meticulous wound care, plastic surgery involvement and prompt treatment of infections at other sites are needed to reduce secondary PJIs.

PJI associated with another site occurred in 27 % of all PJIs, accounting for a high proportion of delayed and late infections. This is presumably because the majority of PJIs associated with wound complication occurred early. These infections demonstrate a persistent risk throughout the lifespan of the implant and are less likely to be influenced by conventional prevention measures at the time of implantation.

Late infections, occurring after 2 years, constituted 20.5 % of all PJIs, which is consistent with observations from longer-term follow-up studies. Jeys et al. (2005) reported that 29.4 % of cases developed PJIs at an average follow-up of 5.8 years (range, 0.25 to 33.6 years). In our study, the median annual risk of PJI beyond 2 years was 1.6 %, presenting a considerable challenge for prevention and highlighting the importance of patient education on long-term risks associated with EPR surgery.

A significant difference in the organism profile was observed between patients with and without wound problems, with a predominance of primary flora among PJIs attributable to primary implantation and Gram-negatives and enterococci among PJIs attributable to wound complications. This difference suggests selection of opportunistic pathogens in the wound is occurring and supports the hypothesis that wound complications are a cause of PJI rather than an early manifestation of PJI in the majority of cases. National and international guidelines recommend that prophylaxis be tailored to the most likely causative organisms. Our data suggest that covering primary flora should be prioritised over Gram-negative organisms when selecting a prophylactic regimen. Among the 19 infections likely acquired at the time of implantation, only two were caused by organisms resistant to the antibiotics administered; potential reasons for this include issues with timing, underdosing or lack of re-dosing.

This study has several limitations. Its retrospective nature may not fully capture the details of wound healing and its timing. Moreover, determining the mode of acquisition based solely on clinical details, such as wound complications, might not always be accurate. We assumed that all PJI in patients with wound complications were acquired due to the wound problem rather than the wound problem being an early manifestation of a PJI. This assumption may not be correct; however, we suggest no study design would be able to reliably distinguish the mode of acquisition of deep infection.

Several potential types of bias are inherent in this study design; we minimised selection bias by including all patients during the study period. Intra-study variability in identifying infection may have occurred; however we minimised detection bias by applying EBJIS criteria consistently across the cohort. The lack of standardised definitions for the full range of wound complications and inherent variability in clinical judgement may pose a risk for misclassification. Some factors potentially limit the broader applicability of our results: the single-centre design may introduce epidemiological bias; however, the large and recently treated cohort strengthens our study. The heterogeneity of this population means the findings may not directly apply to EPRs at specific sites.

Despite these limitations, our study provides new insights into the epidemiology of EPR infections.

## 6 Conclusions

In our study, the majority of PJIs following EPR for musculoskeletal tumours appeared to be due to secondary causes and would not be preventable by antibiotic prophylaxis. Only 26 % were likely to be acquired during the implantation procedure and were predominantly caused by primary flora, suggesting antibiotic prophylaxis should focus specifically on these organisms rather than extending to broader spectrum coverage.

## Data Availability

The datasets used and/or analysed during the current study are available from the corresponding author on reasonable request.
